# Supplementary material for: Predicting the Pathway Involvement of All Pathway and Associated Compound Entries Defined in the Kyoto Encyclopedia of Genes and Genomes
Source: Metabolites. 2024 Oct 27;14(11):582. doi: 10.3390/metabo14110582 (PMC11596622; doi:10.3390/metabo14110582)
Supplement: Supplementary file 1 [file metabolites-14-00582-s001.zip › metabolites-3272988-supplementary.pdf]

Table S1 – Computational Resource Usage of Training the Final Model of Past and Current Data Loading Method.

Data Loading Method	Resource	Unit	Amount
**Old**	CPU Utilization	%	62.6
	GPU RAM	Gigabytes	9.0
	GPU Utilization	%	8.8
	RAM	Gigabytes	22.8
	Real Time	Minutes	978.2
**New**	CPU Utilization	%	98.2
	GPU RAM	Gigabytes	13.2
	GPU Utilization	%	93.7
	RAM	Gigabytes	3.9
	Real Time	Minutes	47.1

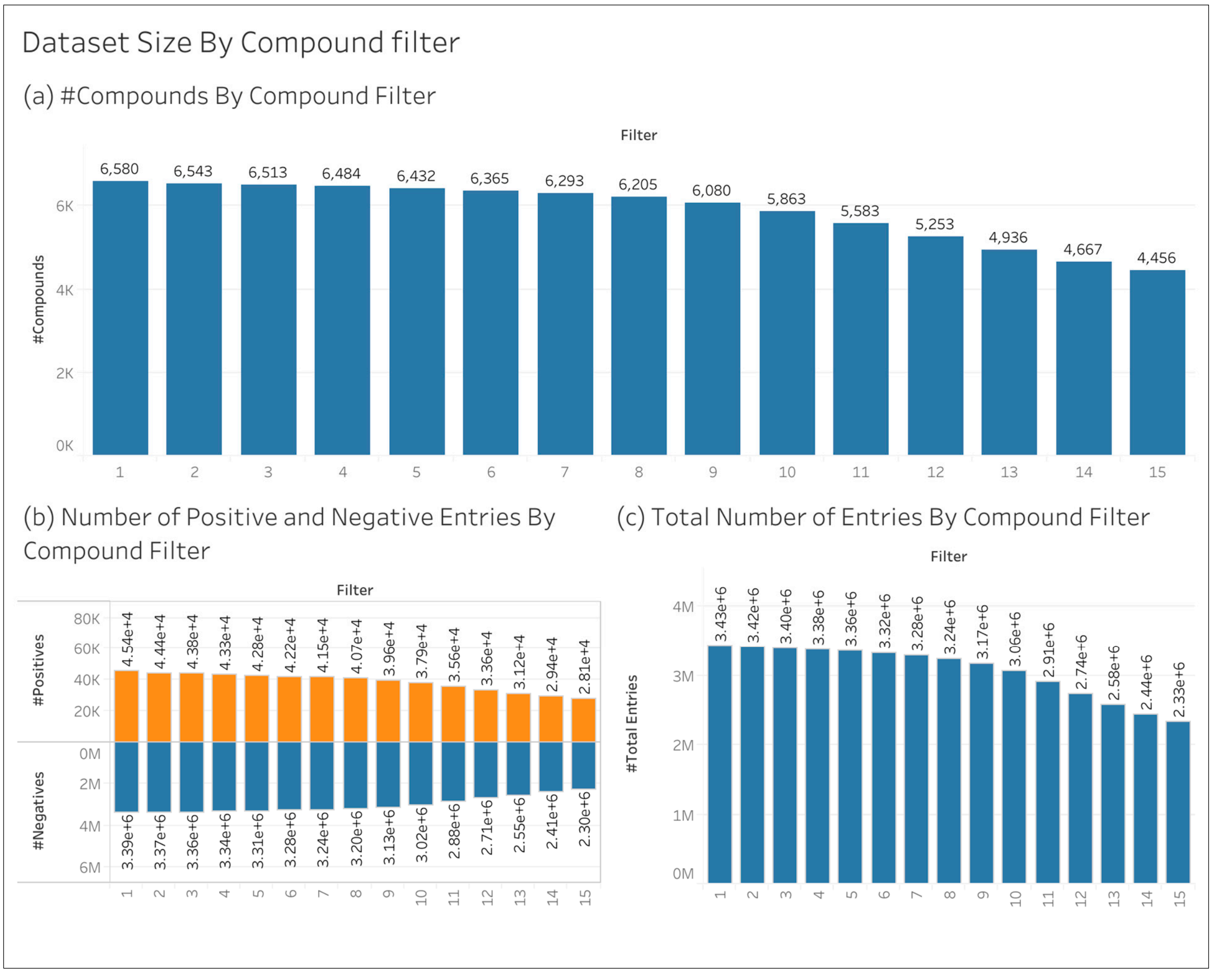

Figure S1 - Size of the Datasets Filtered by Compound Size Based on a Threshold of the Number of Non-hydrogen Atoms.

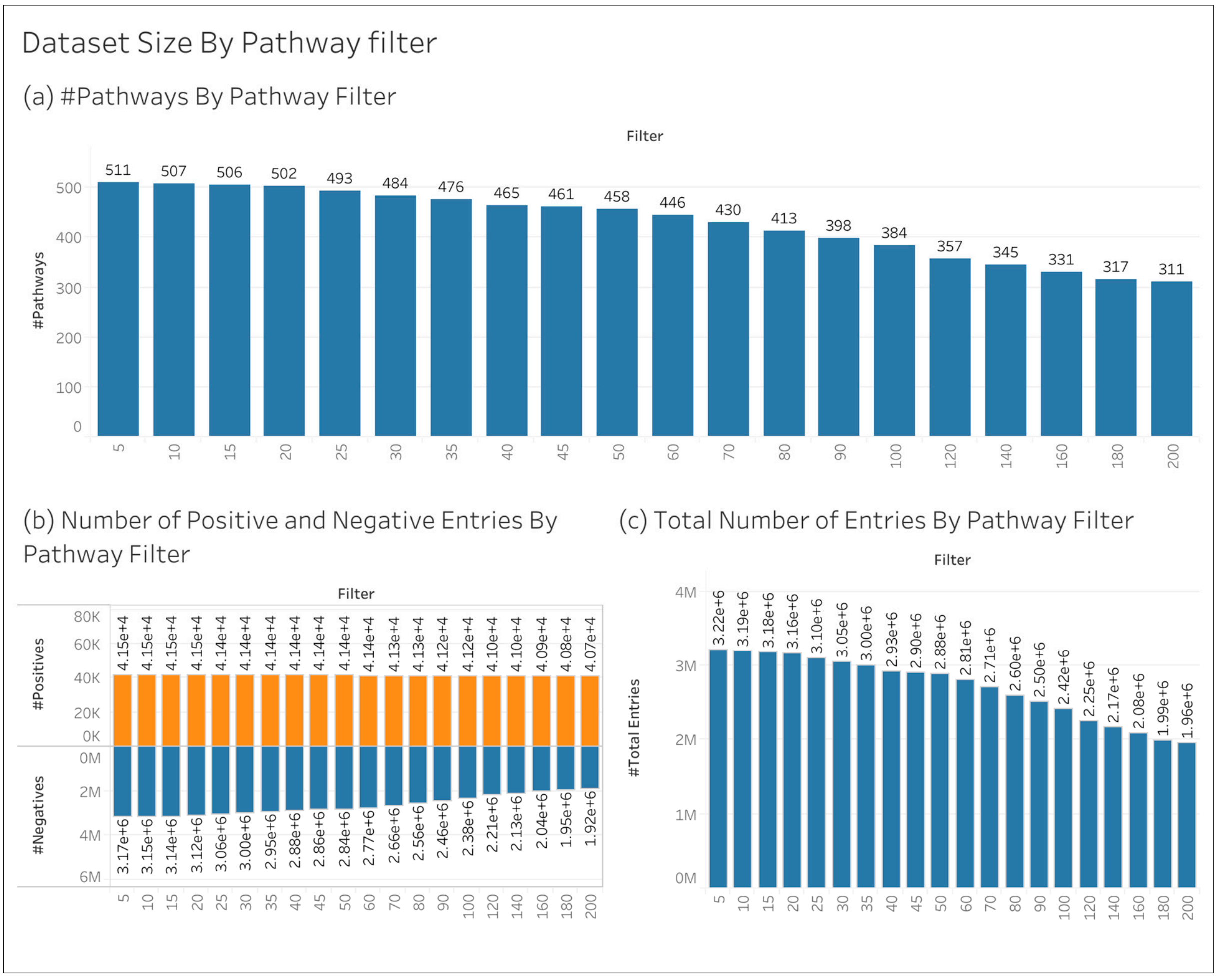

Figure S2 - Size of the Datasets Filtered by Pathway Size Based on a Threshold of the Total Number of Non-hydrogen Atoms Across All the Compounds Associated With Each Pathway.

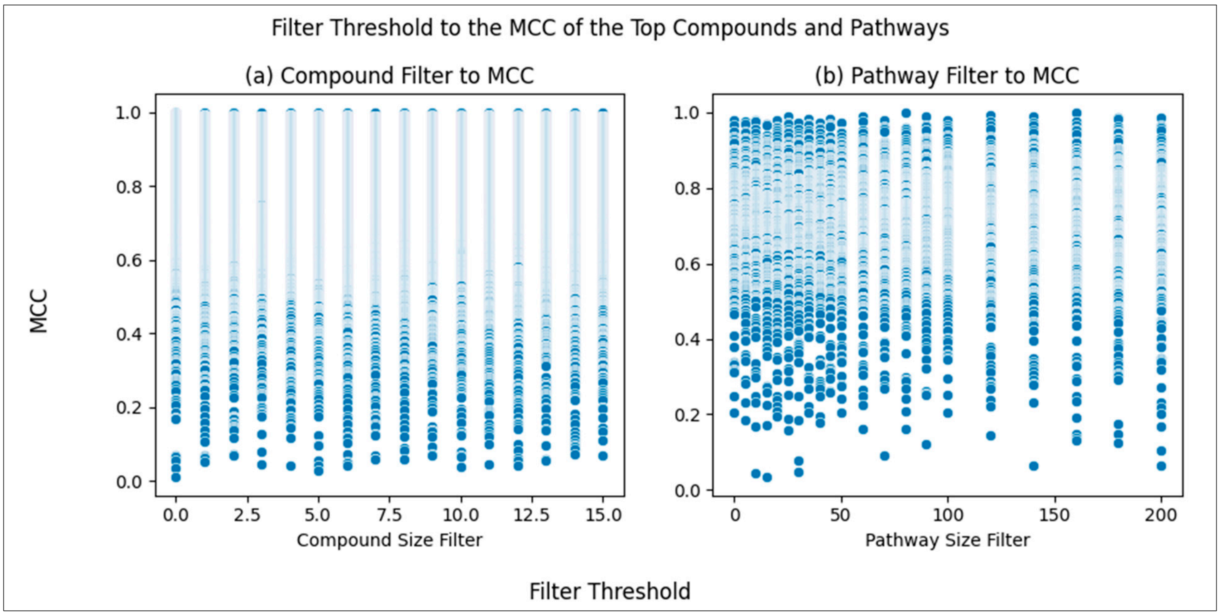

*Figure S3 - Filter Threshold of Number of Non-hydrogen Atoms to the MCCs of the Compounds and Pathways of the Highest Threshold: (**a**) The Compound Size Filter of Each Dataset to the MCCs of the Largest Compounds When Trained On That Dataset; (**b**) The Pathway Size Filter of Each Dataset to the MCCs of the Largest Pathways When Trained On That Dataset.*

Table S2 - Scores for All Metrics by Pathway Hierarchy Levels Included in the Dataset.

Pathway Hierarchy Levels Included	Metric	Mean Score	Median Score	Standard Deviation
**L1, L2, and L3**	Accuracy	0.996	0.996	0.0003
	F1 Score	0.848	0.850	0.0100
	MCC	0.847	0.848	0.0098
	Precision	0.825	0.826	0.0184
	Recall	0.874	0.875	0.0145
	Specificity	0.997	0.997	0.0004
**L2 and L3**	Accuracy	0.996	0.996	0.0004
	F1 Score	0.820	0.823	0.0142
	MCC	0.819	0.821	0.0135
	Precision	0.793	0.797	0.0289
	Recall	0.850	0.850	0.0154
	Specificity	0.997	0.997	0.0005
**L3**	Accuracy	0.996	0.996	0.0003
	F1 Score	0.728	0.731	0.0129
	MCC	0.726	0.729	0.0127
	Precision	0.711	0.713	0.0238
	Recall	0.746	0.747	0.0141
	Specificity	0.998	0.998	0.0003

Table S3 - Pathways With a Null MCC.

Pathway Name	Pathway Size	True positives	True negatives	False positives	False negatives
**path:map04610**	76	0	129,927	0	19
**path:map07053**	26	0	129,277	0	18
**path:map07110**	20	0	129,872	0	36
**path:map07214**	15	0	129,457	0	17

Table S4 - MCC by Pathway Hierarchy Levels Included in the Dataset (preliminary).

Pathway Hierarchy Levels Included	Mean MCC	Median MCC	Standard Deviation
**L1, L2, and L3**	0.822	0.827	0.017
**L2 and L3**	0.799	0.805	0.020
**L3**	0.707	0.710	0.021
